# Application of Mössbauer Spectroscopy for Identification of Iron-Containing Components in Upper Silesian Topsoil Being under Industrial Anthropopressure

**DOI:** 10.3390/ma13225206

**Published:** 2020-11-18

**Authors:** Patrycja Kierlik, Aneta Hanc-Kuczkowska, Marzena Rachwał, Ryszard Męczyński, Izabela Matuła

**Affiliations:** 1 Institute of Materials Engineering, University of Silesia, St. 75 Pułku Piechoty 1A, 41-500 Chorzów, Poland; aneta.hanc@us.edu.pl (A.H.-K.); ryszard.meczynski@us.edu.pl (R.M.); imatula@us.edu.pl (I.M.); 2 Institute of Environmental Engineering, Polish Academy of Sciences, 34 Sklodowska-Curie St., 41-819 Zabrze, Poland; marzena.rachwal@ipis.zabrze.pl

**Keywords:** Mössbauer spectroscopy, qualitative phase analysis, magnetic susceptibility, environmental pollution, topsoil, magnetic separation

## Abstract

The main objective of the presented preliminary study was the identification of iron-containing phases. Iron-containing phases had accumulated in organic topsoil horizons collected from an area that has long been affected by the steel industry and emissions from power plants. X-ray diffraction and Mössbauer spectroscopy methods were used for the determination of the iron-containing mineral phases in topsoil subsamples which, after two-staged separation, varied in terms of magnetic susceptibility and granulometry. The Mössbauer spectra were recorded using paramagnetic and magnetic components, although the latter occurred only in the strongly magnetic fraction. The central part of spectra was fitted by two doublets (D1 and D2), which were identified as aluminosilicates. Simultaneously, the experimental spectra were described using several Zeeman sextets (Z1, Z2, and Z3) corresponding to the occurrence of hematite and magnetite-like phases with iron in tetrahedral and octahedral sites. Identification of magnetic phases in the tested material, including hematite, led to the conclusion that soil contamination in the studied area was presumably caused by emissions from a nearby power plant. Magnetite-like phases with a different iron content detected in topsoil samples could be related to metallurgical and coking processes, reflecting the specificity of the industrial area from which the samples were taken. The specific composition of the iron-containing aluminosilicates also illustrated the intense and long-lasting impact of the steel and coking industries on the studied area.

## 1. Introduction

Intensive industrialization and urbanization are the main reasons for environmental pollution, which has had a serious impact on human health. It is necessary to assess and monitor the environmental hazards caused by industrial emissions as well as to identify contaminants [1,2]. Urban, industrial, and traffic-related emissions are the main source of iron mineral-rich dust, which are byproducts of fossil-fuel combustion and high-temperature technological processes [3,4]. Due to how soils contaminated by the above-mentioned dust and various types of soil sediments are characterized by specific magnetic properties, Mössbauer spectroscopy is needed for a deeper analysis of the impact of anthropogenic pollution on the environment.

Paramagnetic iron minerals are naturally occurring components of soil, rocks, sediments, coal, and other raw materials used in industry. They acquire ferrimagnetic properties as a result of thermal transformations during high-temperature industrial processes. These iron compounds include oxides, sulfides, and hydroxides [5]. The chemical composition of dust from various industries differs depending on the type of fuel used in the production process and the kind of technology used. Iron compounds of industrial dusts are often referred to as technogenic magnetic particles (TMPs). These particles are carriers of metals and metalloids which can be adsorbed onto their surface or incorporated into crystal lattices [6]. Due to the presence of iron compounds in industrial dust, it is possible to determine their presence in the environment with the use of ^57^Fe Mössbauer spectroscopy [7].

Deposition and accumulation of atmospheric particles on different surfaces has been examined by analyzing the various components of the ecosystem (snow [8], street dust [9], industrial dust [10], tree leaves [11]). As opposed to the periodically variable elements of the ecosystem where harmful substances are dispersed, these substances accumulate in the soil [12,13]. Many authors have indicated that the organic layer is the main soil horizon of anthropogenic particle accumulation [14]. Taking into account the long-term impact of human activities on the environment, it is necessary to determine the accumulation of TMP in soils exposed to urban, road-traffic, and industrial emissions.

The main objective of this preliminary study was to determine iron-containing phases, which are accumulated in organic horizons of topsoil collected from an area subjected to the long-term effects of the steel industry and emissions from power plants. In the Upper Silesia agglomeration, there are many areas under strong anthropopressure. The studies presented in this article, which describe the accumulated pollutants in the upper soil layer, are a continuation of our research presented in reference [4], on using Mössbauer spectroscopy to analyze the composition of iron-containing phases in industrial soils (urban soils) exposed to the impact of power plants and coking plants. Many iron-containing phases such as aluminosilicates with Fe^3+^, hydroxide aluminosilicates with Fe^2+^, as well as magnetic phases (magnetite and pyrrhotite) have been identified [4]. Pyrrhotite has been recognized as a characteristic ferromagnetic mineral that is formed during high-temperature pyrolysis of coking coal [5,15]. It is important to continue study aimed at understanding and explaining the mechanism of accumulation of pollution in industrial and post-industrial regions. Therefore, the identification of phases related to the emissions from a steelworks and a power plant will be the goal of this research. Moreover, before the main analysis, an innovative manner of soil separation (two-stage: granulometric and magnetic) was tested in order to obtain extremely different fractions.

## 2. Materials and Methods

The research was carried out in the central part of the Upper Silesian conurbation (southern Poland), the most urbanized, industrialized, and most polluted region of Poland [16]. There were numerous concentrated mines, steelworks, and coke and power plants, which are currently closed for the most part. They, however, have transformed the landscape and produced a lot of waste heaps, related to historical coal and ore mining and processing. Nowadays, it is nearly impossible to find an area in this region that has not been transformed by human activity. As such, soils in this area have been strongly transformed and contaminated [17,18,19,20].

First of all, before soil sampling, magnetic screening using Bartington MS2D susceptibility equipment (Bartington Instruments Ltd., Witney, UK) [21,22] was carried out in the area surrounding a steelworks and a power plant, which are located within ~7 km of each other. On the basis of spatial distribution of magnetic susceptibility (ĸ expressed in 10^−5^ SI units), hotspots (sites with the highest ĸ values) were chosen as the sites for soil collection in the amount of 1–1.5 kg, and the average field-wet bulk topsoil sample was taken from an area of approximately 2 m^2^.

After homogenization, root removal, and exsiccation in the air, samples were grounded and sieved through a 2-mm mesh. Afterwards, each sample was subjected to a two-stage separation: sieve separation and magnetic separation. After the first step, two granulometric fractions, with different grain sizes, I (Ø = 0.05–0.10 mm) and II (Ø = 0.25–0.50 mm), were attained. Both of them were then subjected to magnetic separation with a Frantz isodynamic magnetic separator, which consists of a vibrating chute mounted centrally between the pole pieces of an electromagnet. The chute may be inclined in any direction and the electromagnet current is continuously adjustable from 0 to 1.7 A. The separator divides a sample into two fractions. The strongly magnetic fraction consists of particles, each having a specific susceptibility above a value determined by the settings. The weakly magnetic fraction consists of particles of susceptibility below this value [23]. Two extreme currents, 0.2 A (strongly magnetic fraction denoted as A) and 1.6 A (weakly magnetic fraction denoted as B), were selected for the purpose of this study. As a result, four different fractions (subsamples) were obtained from one topsoil sample.

The subsamples placed in 1 cm^3^ plastic vials were weighed and subjected to volumetric magnetic susceptibility (ĸ) survey. The MFK1 Kappabridge device (Agico Advanced Geoscience Instruments Co., Brno, Czech Republic) was used, and low frequency (976 Hz) and low field intensity (200 A/m) were chosen for standard ĸ measurements at room temperature. Each sample was measured at least five times, and then the mean value was calculated. Afterwards, on the basis of the mean ĸ value and taking into account the weight of the samples, the mass-specific magnetic susceptibility (χ, m^3^/kg) was computed.

Each subsample was subjected to phase composition analysis using the X-ray diffraction method (XRD). Measurements were carried out using an X’Pert Pro Model 3040/60 (Phillips, Eindhoven, Holland) X-ray diffractometer with a copper anode (CuKα − λ = 1.54178 Ǻ) powered by an electric current of 30 mA, voltage of 40 kV, and a curved graphite monochromator to determine the wavelength emitted by the Cu anode. The diffraction patterns were recorded by “step scanning” of 0.04° 2θ steps in the angular range 2θ = 10 ÷ 140°.

Afterwards, the Mössbauer spectroscopy method was used for identification of iron-containing phases in the studied material. A conventional spectrometer working at room temperature with constant acceleration with ^57^Co:Rh source (activity ~50 mCi) in transmission geometry was used for recording the Mössbauer spectra. Metallic iron powder (α-Fe) absorbent was used for velocity and isomer shift calibration of the Mössbauer spectrometer. Samples for Mössbauer analysis were prepared as the absorbents of pressed soil separates without the use of a binder, which could have further disturbed the registration process. Due to the relatively low iron content in the tested material, the spectral registration process was carried out for about 5 days. After each recording of the experimental spectrum, a calibration measurement was performed. The process of deconvolution of experimental spectra was carried out using a dedicated PMOS numerical program [24] with implemented models, allowing for a discrete analysis of experimental spectra and an analysis using the distribution of magnetic fields H (T) and G—full width at a half maximum. The analysis process was carried out in two stages. In the first stage, each spectrum was analyzed separately, while in the second stage, the analysis covered individual series of spectra with common parameters. Numerical analysis included the characteristics of the middle part of the spectrum with the use of paramagnetic components, while the magnetic components were identified on the basis of the Zeeman sextet distribution. As a result of this analysis, a set of components describing the experimental spectra was obtained. The quality parameter of their fit (χ^2^) did not exceed 2, and there were no differences between the curve illustrating the differences in the model and the experimental description, proving the presence of additional components. The mineralogical identification of hyperfine parameters was based on the Mössbauer Mineral Handbook [25].

## 3. Results and Discussion

As expected, the mass-specific magnetic susceptibility varied widely, from 3.6 to 247.6 × 10^−8^ m^3^/kg (Table 1), with the highest values for the finest (Ø = 0.05–0.1 mm) and strongly magnetic (0.2 A) fraction (IA), while the lowest χ was noticed for subsample IB (Ø = 0.05–0.1 mm, 1.6 A), although the value was almost equal to the χ of IIB subsample (χ = 4.0 × 10^−8^ m^3^/kg).

Theoretically, the lowest χ should have the coarser subsample (IIB), but probably the factors other than grain size influenced χ of this topsoil sample, as magnetic properties depend not only on the grain size of magnetic particles but also on their mineralogy and elemental concentration [26,27,28]. The influence of grain size on magnetic susceptibility was evidenced by the almost twice as high value of χ in the case of the finer IA subsample.

**Figure 1:**
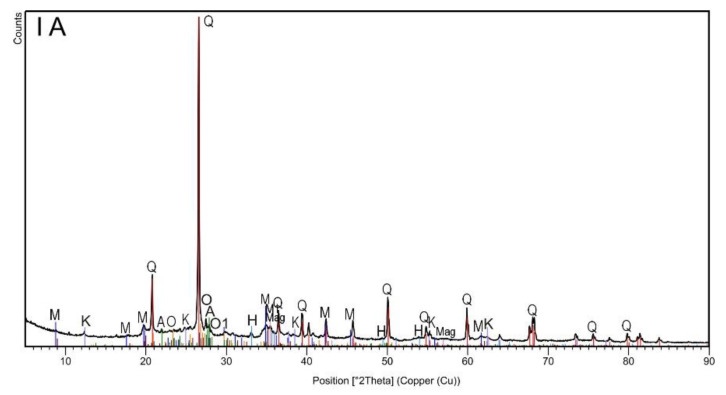

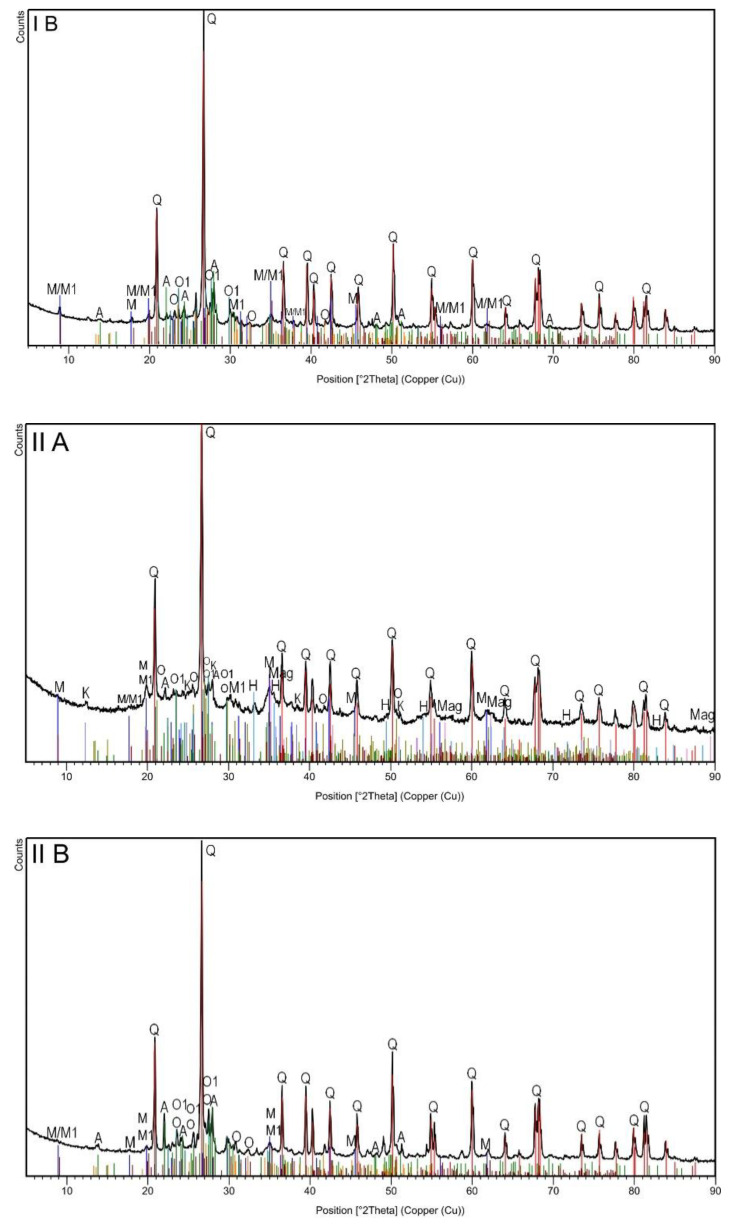
The X-ray diffraction patterns of topsoil subsamples IA, IB, IIA and IIB.

The XRD analysis (Figure 1, Table 2) revealed the multiphase composition of the studied materials of all analyzed samples. In general, six phases (quartz; two types of muscovite; albite and orthoclase with and without Fe) were common for all samples irrespective of the magnetic and grain fraction (Table 2). Since quartz is one of the most common components of rocks and soils, its highest proportion was found in all samples. XRD diffractograms showed major peaks of quartz SiO_2_ (Q: 2θ = 20.8°, 26.7°, 36.5°, 45.8°, 50.3°, 60.1°, 68.2°) [29]. The next quantitative phase was muscovite, which belongs to the minerals from the mica group. Typical muscovite impurities are Fe, Ge, Ti, Li, Na, Ca, Mg, and Fe, which can appear in the crystal structure. Minor modifications to the composition and crystal structure cannot be clearly noticed by phase analysis alone. Several polytypes of this mineral are generally known [30]. In the studied topsoil samples, muscovite was observed in two forms: muscovite-2M1 with additional elements such as Na, Mg, Ti, and Fe (Table 2), and albite-sodium aluminosilicate with an ordered Al-Si distribution. Ca, K, and Mg are the typical impurities of albite, but in the studied samples, such impurities occurred in a rather pure form. The next identified phase was orthoclase, which had epitaxial relationships with albite. Typical orthoclase impurities are Na, Fe, Ba, and Ca, but in this case, only iron was detected. In the case of samples separated by lower current (0.2 A), i.e., strongly magnetic subsamples in both granulometric fractions, the analysis revealed the presence of three additional phases (kaolinite, magnetite, and hematite). The peak at 12.4°, attributed to aluminosilicate plates, corresponds to the basal spacing of kaolinite Si_2_Al_2_O_5_(OH)_4_. Other diffraction peaks could be found at 2θ = 24.9°, 38.7°, and 62.5° [31]. The observed kaolinite was in a 1Md type of structure. Finally, iron oxides (hematite and magnetite) were detected in both strongly magnetic subsamples (IA and IIA). The presence of these iron oxides seemed obvious, especially considering the very high magnetic susceptibility of these subsamples. The broad diffraction peaks at 33.3° in IA and IIA subsamples (Figure 1) could have originated from hematite Fe_2_O_3_, while the peak at 30.0 and 35.8° could have originated from magnetite Fe_3_O_4_ [29].

Summarizing the results of the XRD analysis, it can be stated that only magnetic separation influenced the differentiation of the mineral phases of the studied soils, while grain size did not affect their mineral composition.

**Figure 2:**
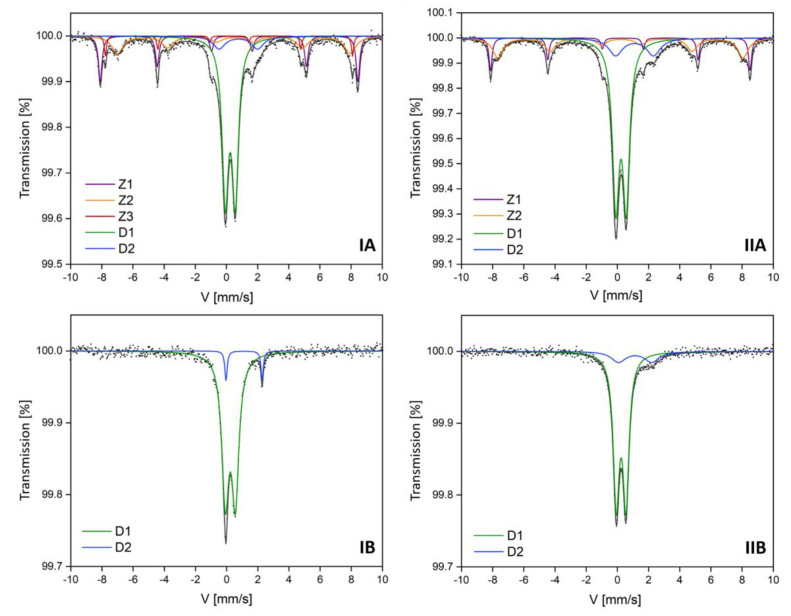
The room temperature Mössbauer spectra of the topsoil subsamples (IA, IIA, IB, IIB). Fitted components are presented on the figure as D1, D2, Z1, Z2, Z3. The *x*-axis of these charts represents velocity (V), while the *y*-axis represents transmission (%). G—full width at half maximum = (0.35–0.40) mm/s.

Mössbauer parameters of the hyperfine interactions are summarized in Table 3 and Table 4. In the central part of the experimental spectra of all subsamples (Figure 2), the presence of paramagnetic components marked as D1 and D2 was found. These paramagnetic components were identified as aluminosilicates with different Fe content. The presence of such components is consistent with the phase identification obtained by the XRD method. D1 doublets were identified as Fe^3+^ aluminosilicates, and D2 doublets were identified as Fe^2+^ aluminum silicate hydroxides [32]. Their percentage contribution was variable. The weakly magnetic subsamples (separated at 1.6 A current) exhibited a higher amount of Fe^3+^ aluminosilicates at 85 and 93% in IB and IIB samples, respectively (Table 4), while the strongly magnetic subsamples IA and IIA (separated at 0.2 A current) contained exactly 51% of Fe^3+^ aluminosilicates (Table 3).

Hyperfine parameters for D1 components may be disturbed by the overlapping of parameters characteristic for magnetite and maghemite nanoparticles, ferrihydrite, or pyrite, the presence of which was not found in the XRD analysis. However, their presence cannot be excluded by analyzing the results contained in the paper of Stevens et al. [25]. The differences between hyperfine parameters for the identified aluminosilicates can also be related to the differences in the chemical composition (iron content, as confirmed by chemical analysis carried out with the SEM-EDS method) of the identified components, which were determined by the ionic forms of iron in these compounds. The spectra of IB and IIB subsamples do not have a magnetic component (no sextet) [33]. This is in agreement with the X-ray diffraction results that revealed the presence of muscovite (M1) and orthoclase (O1), which presumably correspond to aluminosilicates identified by Mössbauer spectra. The proportion of aluminosilicate hydroxide with Fe^2+^ doublet (D2) revealed various dependencies. The magnetic fraction did not influence its content in investigated samples, but the share of D2 component in the coarse granulometric fraction was twice as high as in the smaller one. The percentage of D2 component in iron-containing phase composition of IA and IB (0.05–0.10 mm) was up to 8%, while for IIA and IIB (0.25–0.50 mm) it was up to 15%.

The experimental spectra of strongly magnetic subsamples (IA and IIA) were fitted by Zeeman sextets (Z1, Z2, and Z3). The Z1 component with a hyperfine magnetic field value of 51 T was identified as hematite with a stable share in the sample (19%). Similar results of Mössbauer spectroscopy were obtained for fly and bottom ashes generated in fluidized bed boilers in a Silesian power plant [34]. In fly ash samples, Waanders et al. found the presence of hematite and compounds with Fe^3+^ ions, as well as amorphous Fe^3+^, in glass and muscovite [35]. The similarities in hyperfine parameters with results obtained in the presented work suggest that fly ashes may be a source of pollution in the studied soils. Hematite could be the product of oxidative pyrolysis of the Fe-sulphates and carbonates, but also it can arise during the dehydroxylation of goethite and lepidocrocite [36].

In both magnetic subsamples IA and IIA, which are characterized by high magnetic susceptibility values, the presence of the Z2 component characterized by a hyperfine magnetic field with a value of about 46 T was observed, which can be assigned to the magnetite-like phase (mixed octahedral Fe^3+^ and Fe^2+^). On the other hand, the additional Z3 component, which was only present for the finest fractions (IA) at H = 49 T, could also be identified as magnetite, but with tetrahedral Fe^3+^ [37,38]. Their contribution is rather low (Z2: 16–20%; Z3: only 4%). In the works devoted to the study of metallurgical and coking dusts, similar results of the hyperfine field values were obtained, and it was suggested that the possible occurrence of magnesium ferrite, as the corresponding hyperfine field values for this spinel, is very similar to that of magnetite [3,39]. The results obtained in this study concerning the magnetite-like phase should be discussed in terms of the applied sieve separation used. In finer granulation (IA) magnetite with Fe^2+/3+^ as well as Fe^3+^ was identified, while in larger granulation (IIA) magnetite, only Fe^2+/3+^ was found. Magnetite in mineral form would be fitted with two Zeeman sextets, as confirmed in sample IA, due to the coexistence of Fe^2+^ and Fe^3+^ in the following chemical formula: Fe^2+^Fe^3+^_2_O_4_. Doriguetto et al. [39] established that at 300 K, natural magnetite is represented by two Zeeman sextets. The first sextet with H = 49 T was identified as magnetite with Fe^3+^, while the second sextet with H = 46 T was described as magnetite with Fe^3+/2+^ [9]. Combustion processes used by various industries (including but not limited to the power, fossil-fuel, transport, and coking industries, metallurgy, etc.) may have been the source of magnetite in the topsoil samples.

A greater share of magnetic components was observed in the spectra of subsample IA (41%) in comparison to that of subsample IIA (35%). This difference is not significant, but it corresponds well with the magnetic susceptibility value (χ), which was higher for IA (247.6 × 10^−8^ m^3^/kg) than for the IIA subsample (139.4 × 10^−8^ m^3^/kg). This confirms the assumption that the higher the content of magnetic iron components, the higher the value of soil magnetic susceptibility.

## 4. Summary

Results of the topsoil samples taken from an industrial area revealed their complex phase composition and an occurrence of iron-containing compounds. Subsamples diversified in terms of their magnetic susceptibility and grain size were characterized by different values of hyperfine parameters and thus diversity in characteristic iron-containing phases. Values of magnetic susceptibility corresponded well with the results of Mössbauer spectroscopy analysis. Weakly magnetic subsamples IIA and IIB (separated at 1.6 A current) exhibited larger amounts of paramagnetic components, i.e. aluminosilicates with Fe^3+^ (85–93%), while the strongly magnetic subsamples IA and IIA (separated at 0.2 A current) contained exactly 51% of aluminosilicates with Fe^3+^. The share of aluminosilicates with Fe^2+^ doublet (D2 component) in the coarse granulometric fraction (IIA and IIB: 0.25–0.5 mm) was twice as high as in the finer topsoil subsamples (IA and IB: 0.05–0.1 mm). Magnetic components (Z1, Z2, and Z3) were identified as hematite and magnetite, with iron in tetrahedral as well as octahedral sites, but only for strongly magnetic separates (IA and IIA). The identification of hematite in Mössbauer spectra analysis led to the conclusion that soil contamination in the studied area could be caused by emissions from power plants, while occurrence of magnetite-like phases in topsoil samples could be related to metallurgical and coking processes.

## Figures and Tables

**Table 1:** Mass-specific magnetic susceptibility (χ) of topsoil subsamples after sieve and magnetic separation.

Topsoil Subsample	Grain Size (mm)	Current Intensity (A)	χ (×10^−8^ m^3^/kg)
IA (strongly magnetic)	0.05–0.1	0.2	247.6
IIA (strongly magnetic)	0.25–0.5	0.2	139.4
IB (weakly magnetic)	0.05–0.1	1.6	3.6
IIB (weakly magnetic)	0.25–0.5	1.6	4.0

**Table 2:** The identified phases in topsoil subsamples after sieve and magnetic separation (presence of particulate phases in sample was marked as “x”).

Identified Phase		Topsoil Subsample			
Chemical Formula	Mineral (Symbol)	IA	IIA	IB	IIB
SiO_2_	Quartz (Q)	x	x	x	x
H_2_KAl_3_(Si0_4_)_3_	Muscovite (M)	x	x	x	x
K_0.60_Na_0.37_Mg_0.06_Ti_0.02_Fe_0.10_Al_2.81_Si_3.03_O_10_(OH)_2_	Muscovite -2M1 (M1)	x	x	x	x
NaAlSi_2_O_8_	Albite (A)	x	x	x	x
Si_2_ Al_2_O_5_(OH)_4_	Kaolinite (K)	x	x		
KAlSi_3_O_8_	Orthoclase (O)	x	x	x	x
K(Al,Fe)Si_2_O_8_	Orthoclase (O1)	x	x	x	x
Fe_3_O_4_	Magnetite (Mag)	x	x		
Fe_2_O_3_	Hematite (H)	x	x		

**Table 3:** Fitted Mössbauer parameters for spectra of samples IA and IIA. IS—isomer shift (with reference to metallic iron), QS—quadrupole splitting, H—magnetic hyperfine filed, A—relative area fraction with respect to whole fitted spectrum. Doublet compounds have been described as D1 and D2 and sexted compounds have been described as Z1, Z2, and Z3.

Subsample	IA				IIA				Iron-Containing Phase
Component	IS	QS	H	A	IS	QS	H	A	
	[mm/s]	[mm/s]	[T]	[%]	[mm/s]	[mm/s]	[T]	[%]	
D1	0.35	0.64	0	51	0.33	0.67	0	51	Aluminosilicates with Fe^3+^
D2	0.86	2.47	0	8	1.24	2.4	0	14	Aluminosilicates with Fe^2+^
Z1	0.36	−0.18	51	17	0.36	−0.16	51	19	Hematite
Z2	0.61	−0.08	46	20	0.5	0.05	46	16	Magnetite
Z3	0.27	−0.02	49	4	-	-	-	-	

ΔIS = ± 0.01 mm/s; ΔQS = ± 0.01 mm/s; ΔH = ± 1 T.

**Table 4:** Fitted Mössbauer parameters for spectra of samples IB and IIB, where doublet compounds have been described as D1 and D2. IS—isomer shift (with reference to metallic iron), QS—quadrupole splitting, A—relative area fraction with respect to whole fitted spectrum.

Subsample	IB			IIB			Iron-Containing Phase
Component	IS	QS	A	IS	QS	A	
	[mm/s]	[mm/s]	[%]	[mm/s]	[mm/s]	[%]	
D1	0.34	0.65	93	0.34	0.62	85	Aluminosilicates with Fe^3+^
D2	1.22	2.32	7	1.24	2.29	15	Aluminosilicates with Fe^2+^

ΔIS = ±0.01 mm/s; ΔQS = ±0.01 mm/s.
